# The Use of Portable, Very Low-field (0.064T) MRI to Image Cochlear Implants: Metallic Image Artifact in Comparison to Traditional, Stationary 3T MRI

**DOI:** 10.1097/ONO.0000000000000049

**Published:** 2024-03-07

**Authors:** Christopher C. Munhall, Donna R. Roberts, Robert F. Labadie

**Affiliations:** 1 Department of Otolaryngology—Head and Neck Surgery, Medical University of South Carolina, Charleston, South Carolina; 2 Department of Radiology, Medical University of South Carolina, Charleston, South Carolina.

**Keywords:** Cochlear implant, MRI, Imaging, Image artifact, Low-field MRI

## Abstract

**Objective:**

To assess image artifact when imaging a cochlear implant (CI) with a conventional 3T MRI machine compared with a very low-field (0.064T) MRI.

**Patients:**

None.

**Intervention:**

Diagnostic study.

**Main Outcome Measure:**

Image artifact size associated with the CI affixed to an MRI phantom at very low-field 0.064T MRI versus 3T MRI.

**Results:**

The longest diameter of the image artifact was 125 mm for the 3T MRI and 86 mm for the 0.064T MRI, representing 45% longer image artifact generated in the 3T MRI. The actual volume of the imaging phantom was 1371 cm^3^. The volume of the image artifact was measured as 379 cm^3^ in the 3T MRI, representing a loss of 27.6% of the actual volume of the imaging phantom. The volume of image artifact was measured as 170 cm^3^ in the 0.064T MRI, representing a loss of 12.4% of the phantom volume.

**Conclusions:**

3T MRI had better image quality. This result was not surprising given that larger magnetic field strength is known to provide higher resolution. There was 15% less image artifact generated in the very low-field MRI machine compared with a conventional 3T device. And there was also subjectively increased distortion of the imaging phantom at 3T MRI compared with the 0.064T MRI. With minimized safety concerns and a much lower cost than conventional 3T machines, very low-field scanners may find expanded clinical uses. This preclinical study explores the potential utility of very low-field MRI in scanning CI recipients.

Technological advances in cochlear implant (CI) technology now allow for 3T MRI of the brain without removal of the CI magnet (1). While a huge advance in the technology, at higher field strength MRI metallic artifact associated with the CI disrupts the magnetic field of the scanner distorting the images and results in the CI and surrounding structures being nonvisible on MRI. Simultaneous technological advances in MRI have resulted in very low-field (0.064T) portable MRI scanners thereby reducing the concern for ferrous-containing metal restrictions near the scanner with the intent of enabling imaging within emergency rooms, intensive care units, and potentially ambulances/vans (2,3) to allow rapid diagnosis at point of care. Theoretically, because the scanner is low magnetic field, the potential disruption of the magnet field and subsequent metallic artifact would be less than for high-field scanners. This pilot study sought to assess image artifact when imaging a CI with a conventional 3T MRI machine compared with an Food and Drug Administration-cleared, portable, very low-field (0.064T) MRI.

## MATERIALS AND METHODS

This pilot study assessed image artifact for a CI externally affixed to an MRI phantom. The primary outcome measure of this study was the image artifact size associated with the CI at very low-field, portable MRI versus 3T MRI.

An Advanced Bionics HiRes Ultra 3D CI (Food and Drug Administration cleared for use at 3T MRI) was affixed to an MRI phantom (a cylindrical fluid-filled object routinely used to assess scanner quality) using hot-melt adhesive and self-adhering wrap. Images were obtained in both a novel, very low-field 0.064T MRI machine, the Hyperfine Swoop (Hyperfine Inc.; Guilford, CT) and the Siemens 3T Prisma^fit^ (Siemens Medical Solutions, Malvern, PA). Computed tomography scans (Xoran XCAT; Xoran Technologies LLC; Ann Arbor, MI) were obtained between imaging in each of the MRI machines to verify consistent CI positioning on the imaging phantom. The dimensions and overall volume of imaging phantom not visualized due to distortion/artifact were calculated using a free, open-source, image-processing software, 3D slicer (slicer.org). Volume renderings of the image sequences were created in 3D slicer using the thresholding tool (a value-based method to filter out image data) followed by manual, hand-drawn adjustments to delineate phantom borders. The volumes of the imaging phantom detected were compared with the true volume of the imaging phantom to determine the extent of distortion in both the 3T MRI and the 0.064T MRI machines.

## RESULTS

The longest diameter of the image artifact was 125 mm for the 3T MRI and 86 mm for the 0.064T MRI, representing 45% longer image artifact generated in the 3T MRI. The artifact measured from surface to maximum depth into the imaging phantom was 63 mm for the 3T MRI and 65 mm for the 0.064T MRI. The actual volume of the imaging phantom was 1371 cm^3^. The volume of the image artifact was measured as 379 cm^3^ in the 3T MRI, representing a loss of 27.6% of the actual volume of the imaging phantom. The volume of image artifact was measured as 170 cm^3^ in the 0.064T MRI, representing a loss of 12.4% of the phantom volume.

**FIG. 1.**
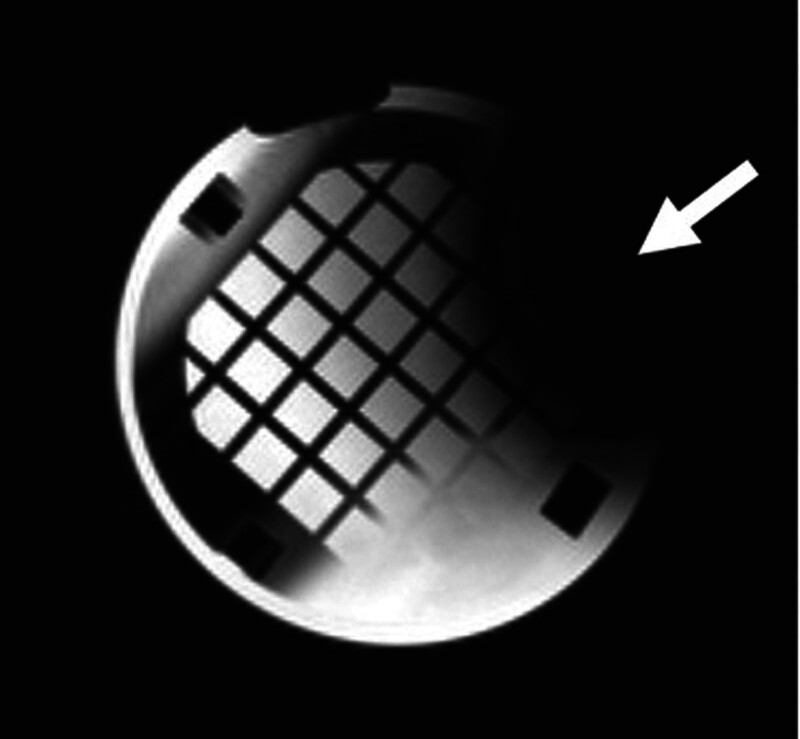
T2 axial image obtained of the imaging phantom in the 3T MRI with moderate image void artifact present (arrow).

**FIG. 2.**
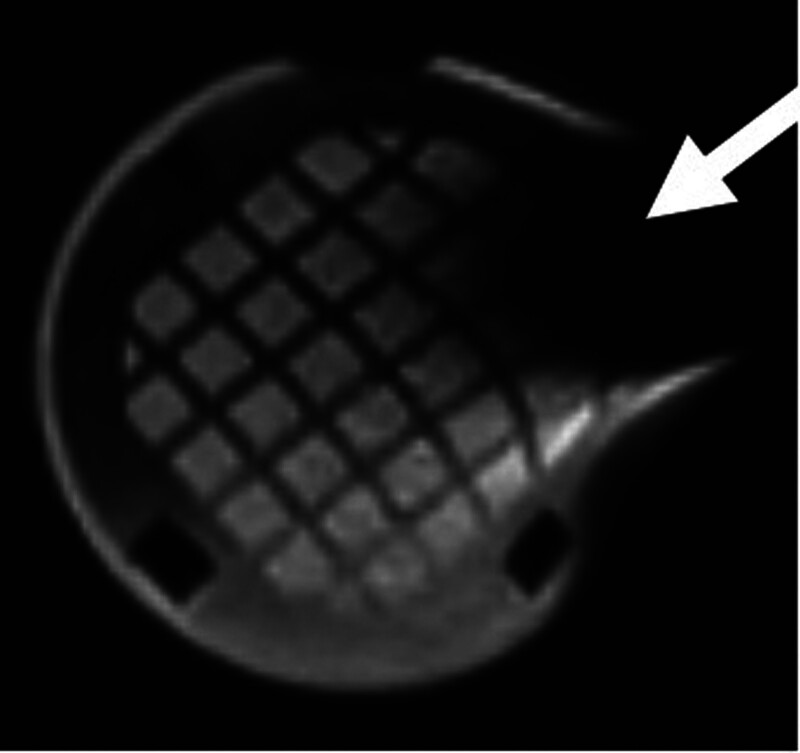
T2 axial image obtained of the imaging phantom in the 0.064T MRI with moderate image void artifact present (arrow).

**FIG. 3.**
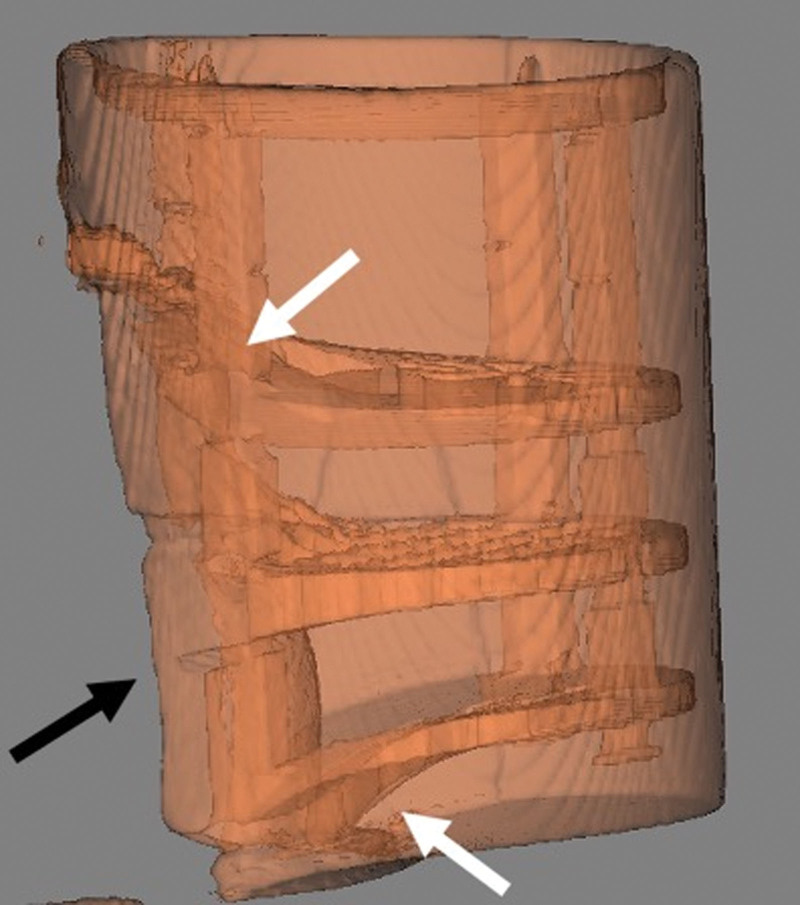
Transparent 3D rendering of the phantom in the 3T MRI with large image void (black arrow) and image distortion (white arrows) generated by the cochlear implant.

**FIG. 4.**
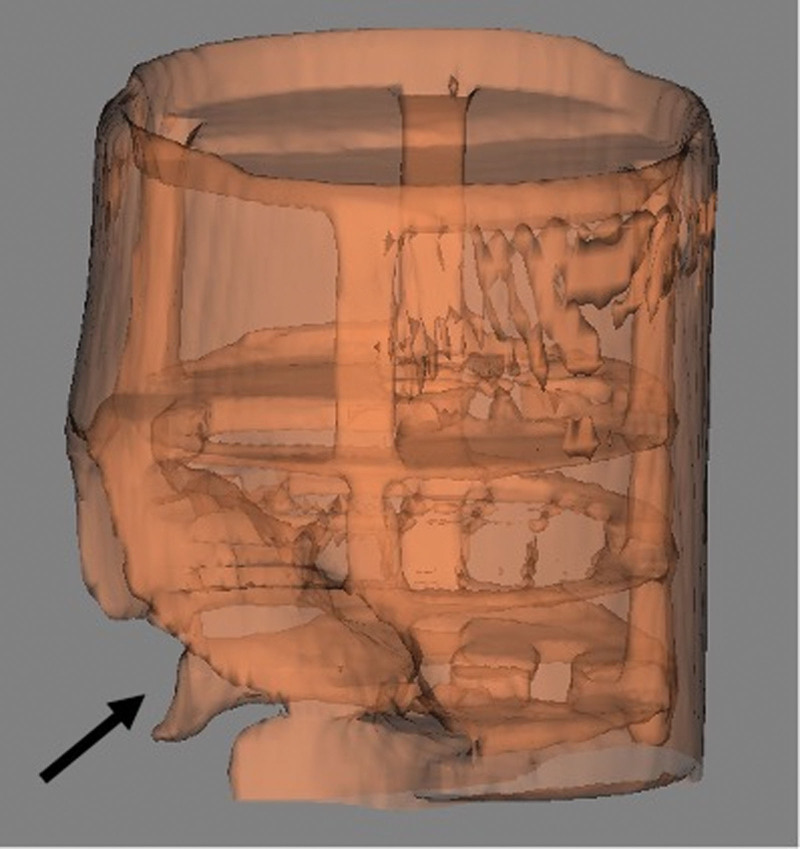
Transparent 3D rendering of the phantom in the 0.064T MRI with moderate image void (arrow) generated by the cochlear implant.

Image quality was subjectively better in the conventional 3T MRI as seen in the axial view of the phantom in Figure 1 compared with image quality obtained in the 0.064T MRI shown in the axial view of the phantom in Figure 2. Objective image quality was determined using paired circular targets present on the imaging phantom to assess image resolution. The 3T MRI demonstrated superior image resolution as the smallest 2 mm targets were clearly identified, while these 2 mm targets were not clearly discernible in the 0.064T MRI. While image quality was better in the 3T machine, there was subjectively more distortion of the imaging phantom as seen in 3D renderings from the 3T MRI (Fig. 3) compared with the 0.064T MRI (Fig. 4).

## DISCUSSION

(1) 3T MRI had better image quality. This result was not surprising given that larger magnetic field strength is known to provide higher resolution. The 0.064T MRI attempts to overcome lower magnet strength by utilizing artificial neural network-driven deep learning algorithms during the gridding step (which involves converting data from the spatial frequency domain to the image domain) and the denoising step (a postprocessing step in which signal noise is removed from the final image) to reduce image noise and blurring, thereby optimizing overall image quality (4). Despite these steps, the image resolution was still noticeably diminished in the very low-field scanner, and some research regarding machine learning reconstruction in MRI has also noted the possibility for hallucinatory effects. (2) There was 15% less image artifact generated in the very low-field MRI machine compared with a conventional 3T device and subjectively increased distortion of the imaging phantom at 3T MRI compared with the 0.064T MRI. It is unclear whether decreased image artifact equates to a clinically meaningful difference in evaluating lateral skull base pathologies in CI recipients; however, it should be noted that the limited image resolution of the 0.064T MRI at 2 mm noted above would limit identification of small areas of pathology even outside areas of image artifact or of the contralateral lateral skull base. (3) Portable, very low-field MRI could offer potential utility in screening for intracranial pathology in CI recipients. With a much lower field 0.064T magnet, typical MRI safety concerns (5) regarding everyday metallic objects (eg, glasses, keys, along with earrings or other body piercings) are minimized compared with 1.5T or 3T scanners (6) and such objects can be brought close to the 0.064T MRI. Although newer generation CI magnets are approved for 3T MRI with greatly reduced rates of adverse effects, risk of demagnetization of the internal CI magnet (7) and heat generated from interaction between the electromagnetic field and CI (8) might both be reduced with lower magnetic field strength. These attributes make the use of a very low-field MRI scanner attractive as a tool for screening CI recipients who need a brain MRI, although feasibility studies for this device (9–11) to date have not evaluated clinical utility in evaluating lateral skull base conditions. (4) Given a price point of $50,000 (2), very low-field portable scanners could provide a cost-effective alternative to conventional MRI in certain health care settings. Toward this end, we have embarked upon an imaging study of CI recipients to determine the optimal imaging protocol at very low-field MRI, which minimizes metallic artifact and image distortion while allowing for the best visualization of intracranial pathologies.

## FUNDING SOURCES

The scanner utilized in this study was provided by Hyperfine (Swoop®, Hyperfine, Inc., Guilford, CT).

## CONFLICT OF INTEREST STATEMENT

None declared.

## DATA AVAILABILITY STATEMENT

The datasets generated during and/or analyzed during the current study are not publicly available but are available from the corresponding author on reasonable request.
